# Cardiac remodeling and arrhythmogenesis are ameliorated by administration of Cx43 mimetic peptide Gap27 in heart failure rats

**DOI:** 10.1038/s41598-020-63336-6

**Published:** 2020-04-23

**Authors:** Claudia M. Lucero, David C. Andrade, Camilo Toledo, Hugo S. Díaz, Katherin V. Pereyra, Esteban Diaz-Jara, Karla G. Schwarz, Noah J. Marcus, Mauricio A. Retamal, Rodrigo A. Quintanilla, Rodrigo Del Rio

**Affiliations:** 1 Laboratory of Cardiorespiratory Control, Department of Physiology, Faculty of Biological Sciences, Pontificia Universidad Católica de Chile, Santiago, Chile; 2 Institute of Biomedical Sciences, Universidad Autónoma de Chile, Santiago, Chile; 3 Centro de Investigación en Fisiología del Ejercicio, Facultad de Ciencias, Universidad Mayor, Santiago, Chile; 4 Centro de Envejecimiento y Regeneración (CARE-UC), Pontificia Universidad Católica de Chile, Santiago, Chile; 5 Department of Physiology and Pharmacology, Des Moines University, Des Moines, IA USA; 6 Universidad del Desarrollo, Centro de Fisiología Celular e Integrativa, Clínica Alemana Facultad de Medicina, Santiago, Chile; 7 Centro de Excelencia de Biomedicina de Magallanes (CEBIMA), Universidad de Magallanes, Punta Arenas, Chile

**Keywords:** Physiology, Cardiovascular biology, Cardiac hypertrophy, Cardiovascular diseases

## Abstract

Alterations in connexins and specifically in 43 isoform (Cx43) in the heart have been associated with a high incidence of arrhythmogenesis and sudden death in several cardiac diseases. We propose to determine salutary effect of Cx43 mimetic peptide Gap27 in the progression of heart failure. High-output heart failure was induced by volume overload using the arterio-venous fistula model (AV-Shunt) in adult male rats. Four weeks after AV-Shunt surgery, the Cx43 mimetic peptide Gap27 or scrambled peptide, were administered via osmotic minipumps (AV-Shunt_Gap27_ or AV-Shunt_Scr_) for 4 weeks. Cardiac volumes, arrhythmias, function and remodeling were determined at 8 weeks after AV-Shunt surgeries. At 8^th^ week, AV-Shunt_Gap27_ showed a marked decrease in the progression of cardiac deterioration and showed a significant improvement in cardiac functions measured by intraventricular pressure-volume loops. Furthermore, AV-Shunt_Gap27_ showed less cardiac arrhythmogenesis and cardiac hypertrophy index compared to AV-Shunt_Scr_. Gap27 treatment results in no change Cx43 expression in the heart of AV-Shunt rats. Our results strongly suggest that Cx43 play a pivotal role in the progression of cardiac dysfunction and arrhythmogenesis in high-output heart failure; furthermore, support the use of Cx43 mimetic peptide Gap27 as an effective therapeutic tool to reduce the progression of cardiac dysfunction in high-output heart failure.

## Introduction

Arrhythmias, or heart rate disruptions, are closely linked to the development of cardiac pathologies and constitute one of the main predictors of morbidity and mortality associated with heart failure disease^1^. Interestingly, the prevalence of atrial fibrillation (AF) in heart failure patients is ~30%, increasing with the severity of cardiac disease according to the New York Heart Association [NYHA] functional class^2–4^. The severity of AF is strongly associated with an increase in hospital readmissions due to decompensation^1^. Similarly, ventricular fibrillation, a kind of ventricular arrhythmia (VA), is considered one of the main contributors to sudden cardiac death in heart failure patients, contributing to more than 50% of all cardiovascular deaths in this population^5–7^. Unfortunately, the current treatments focused on reducing arrhythmogenesis are not fully successful due to the negative inotropic effect^7^, that many of them have and that could cause a worsening of the HF, and because of the complexity of events that are involved in the genesis of arrhythmia^6,7^. It has been proposed that disruption of electrical properties in the heart, such as intercellular uncoupling between cardiomyocyte and cardiac fibrosis, both are considerate as two of the most important arrhythmogenic substrates associated with HF^8^. Therefore, if these factors are present, the increase in the severity of the type of arrhythmia and consequently, the worsening of cardiac function would be caused.

Gap junctions (GJ) are channels that allow electrical coupling between contiguous cardiomyocytes^9^. These structures are formed by connexins (Cx), being the isoform 43 (Cx43) the most constitutively expressed in cardiac tissue^9^. In addition to GJ, Cx also form hemichannels (HCs), which allow for the exchange of ions and small metabolites of low molecular weight between the inside of the cardiomyocyte and the extracellular milieu^9,10^. In a physiological state, GJs are in open conformation while HCs are usually closed; however, this mechanism is altered in pathophysiological conditions, where HCs are more likely to be open while the permeability of GJ is restricted^10,11^. Alterations in Cx43 function, expression, phosphorylation states and localization are present in several human cardiomyopathies and these are strongly correlated with the incidence of cardiac arrhythmias and cardiac dysfunction^12–14^. Indeed, patients with heart disease, including heart failure showed an increase in Cx43 localized in lateral walls of cardiomyocytes, forming HCs, and a reduction in Cx43 located at intercalated discs, in shape of GJs^12,15–17^. In addition, it has been shown that sympathetically-induced cardiac arrhythmias in a Duchenne muscular dystrophy model are partly mediated by Cx43 HCs since HCs blockers reduces the number of arrhythmic episodes^16^.

Considering that heart failure is associated with both cardiac arrhythmias and conformational changes of Cx43 in the heart, and that Cx43 blockade decreases sympathetic-mediated cardiac arrhythmias in non-ischemic dystrophic hearts, we hypothesized that Cx43 mimetic peptide Gap27 will improve cardiac function and reduce arrhythmogenesis in non-ischemic heart failure. Accordingly, we studied the effects of chronic administration of Cx43 mimetic peptide Gap27 on the progression of cardiac dysfunction, incidence of cardiac arrhythmias, cardiac function and cardiac remodeling in rats with high-output heart failure (AV-shunt) a well-characterized model of non-ischemic heart disease with neurohumoral activation, sympatho-excitation and cardiac dilation^18–20^. We found that heart failure rats treated with Gap27 showed a marked decrease in the progression of cardiac function deterioration, in cardiac arrhythmogenesis and cardiac hypertrophy compared to vehicle-treated heart failure rats. These data strongly support the notion that Cx43 play a pivotal role in the progression of cardiac dysfunction and arrhythmogenesis in heart failure condition; in addition, our data support that Cx43 mimetic peptide Gap27 may have salutary effect in high-output heart failure.

## Results

### Chronic Gap27 administration delays the progression of cardiac LV dilation in AV-Shunt rats

**Figure 1:**
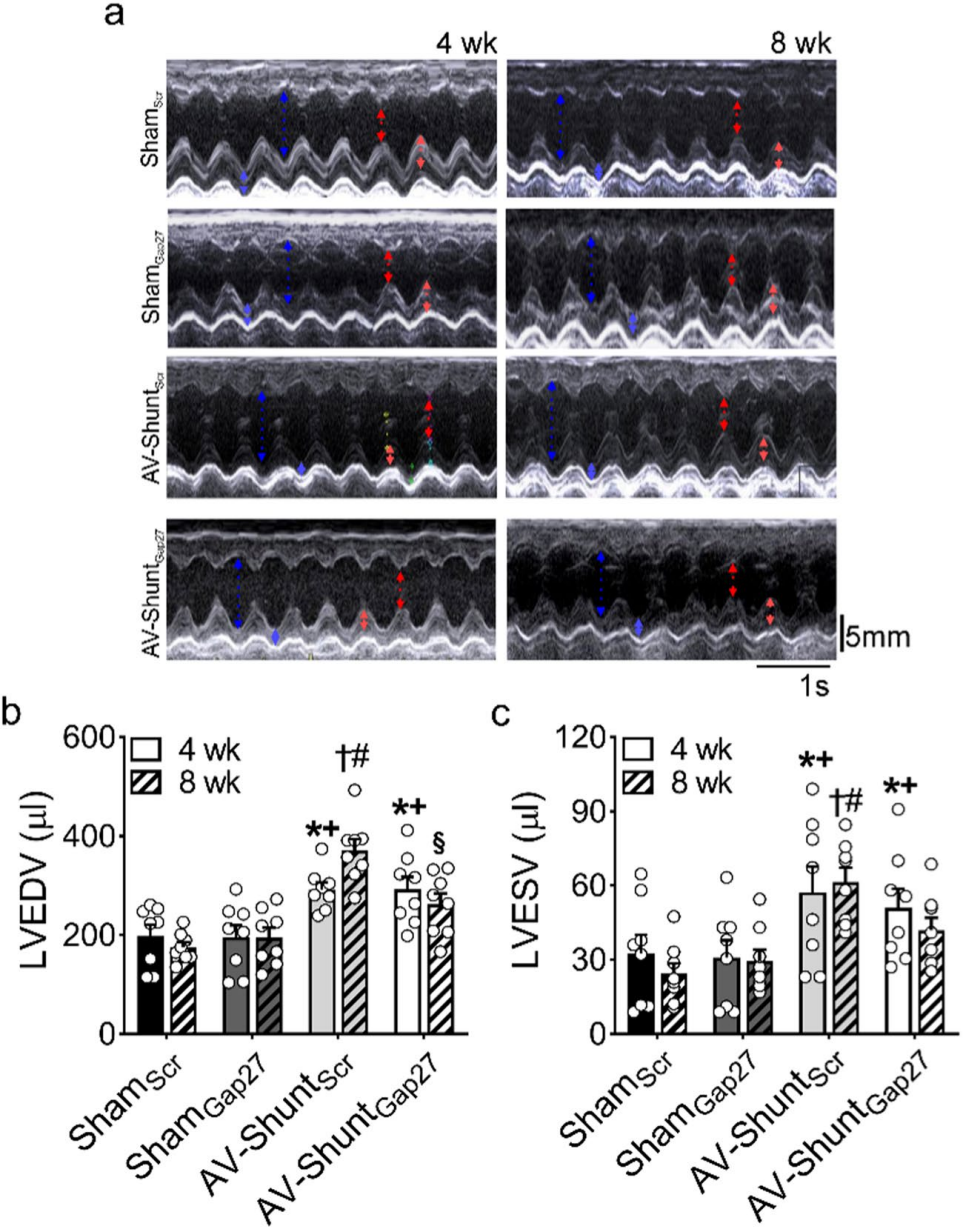
Effect of Gap27 on echocardiographic parameters in AV-Shunt rats. **a** Representative echocardiographic image of the left ventricle from one Sham_Scr_ rat, one Sham_Gap27_ rat, one AV-Shunt_Scr_ rat and one AV-Shunt_Gap27_ rat at 4^th^ and 8^th^ weeks post-AV-Shunt (before and after treatment with Gap27, respectively). Summary data of LVEDV **b** and LVESV **c** parameters. Values are shown as mean ± SEM. Data were analysed with 2-way ANOVA followed by Sidak post hoc analysis. *p < 0.05 vs. Sham_Scr_ 4 wk; ^+^p < 0.05 vs^.^ Sham_Gap27_ 4 wk; ^†^p < 0.05 vs. AV-Shunt_Scr_ 4 wk; ^‡^p < 0.05 vs. AV-Shunt_Gap27_ 4 wk; ^#^p < 0.05 vs. Sham_Scr_ 8 wk; ^§^p < 0.05 vs. AV-Shunt_Scr_ 8 wk. n = 8 animals per group.

AV-Shunt_Scr_ rats exhibited a progressive increase in cardiac volumes at 8^th^ week compared to 4^th^ week. Left ventricular end diastolic volume (LVEDV:370.7 ± 22.7 vs. 291.9 ± 14.8 µl, p < 0.05 AV-Shunt_Scr_ 8^th^ week vs AV-Shunt_Scr_ 4^th^ week, respectively) (Fig. 1a,b) and left ventricular stroke volume (LVSV: 309.3 ± 20.7 vs. 234.6 ± 11.8 µl, p < 0.05 AV-Shunt_Scr_ 8^th^ week vs AV-Shunt_Scr_ 4^th^ week, respectively) (Table 1) were significantly increased compared to Sham rats. In addition, AV-Shunt_Scr_ rats showed a significant increase in left ventricular end systolic volume at 4^th^ weeks (LVESV: 57.25 ± 10.31 vs. 32.56 ± 7.53 µl, p < 0.05, AV-Shunt_Scr_ 4^th^ week vs Sham_Scr_ 4^th^ week, respectively) and 8^th^ weeks compared to Sham_Scr_ animals (LVESV: 61.40 ± 5.78 vs. 24.26 ± 4.25 µl, p < 0.05, AV-Shunt_Scr_ 8^th^ week vs Sham_Scr_ 8^th^ week, respectively) (Fig. 1c). Chronic Gap27 administration decreases the progression of cardiac deterioration between the 4^th^ and 8^th^ weeks. Indeed, we found that LVEDV and LVSV in AV-Shunt_Gap27_ remained unchanged compared to the values obtained at 4^th^ week post-HF induction (Fig. 1b and Table 1). No positive effects were found in LVESV (41.8 ± 5.1 vs. 61.4 ± 5.8 µl, AV-Shunt_Gap27_ 4^th^ week vs AV-Shunt_Scr_ 8^th^ week, respectively). Similarly, no effects of Gap27 on echocardiography parameters were found in Sham rats (Fig. 1 and Table 1).

**Table 1:** Echocardiographic parameters at 4^th^ and 8^th^ week post shunt surgery.

	Sham_Scr_ (n = 8)		Sham_Gap27_ (n = 8)		AV-Shunt_Scr_ (n = 8)		AV-Shunt_Gap27_ (n = 8)	
	4 wk	8 wk	4 wk	8 wk	4 wk	8 wk	4 wk	8 wk
LVESD (mm)	2.8 ± 0.3	2.5 ± 0.2	2.7 ± 0.3	2.8 ± 0.2	3.6 ± 0.3*	3.8 ± 0.1#	3.4 ± 0.2	3.2 ± 0.2
LVEDD (mm)	6.2 ± 0.3	5.9 ± 0.2	6.1 ± 0.4	6.2 ± 0.3	7.4 ± 0.2*	8.3 ± 0.2‡#	7.4 ± 0.3*	7.0 ± 0.3#§
LVESPW (mm)	3.3 ± 0.2	3.6 ± 0.2	3.0 ± 0.1	3.8 ± 0.2	2.7 ± 0.2	3.0 ± 0.2	2.9 ± 0.3	3.5 ± 0.2
LVEDPW (mm)	2.7 ± 0.2	3.1 ± 0.3	2.4 ± 0.2	3.1 ± 0.2	2.0 ± 0.1	2.2 ± 0.3#	2.2 ± 0.2	3.0 ± 0.2§
LVSV (µl)	166.4 ± 16.5	151.2 ± 7.9	164.2 ± 17.7	165.3 ± 16.0	234.6 ± 11.8*	309 ± 21‡#	241.3 ± 18.2*	220 ± 18#§
LVFS (%)	56.1 ± 3.0	57.5 ± 2.3	56.6 ± 2.4	55.4 ± 1.5	52.0 ± 3.4	54.3 ± 1.6	53.7 ± 1.4	54.7 ± 1.0
LVEF (%)	85.0 ± 2.7	86.5 ± 1.9	85.7 ± 1.9	85.1 ± 1.2	80.8 ± 3.1	83.3 ± 1.4	83.1 ± 1.3	84.2 ± 0.9
BW (g)	461 ± 36	527 ± 38	484 ± 45	527 ± 31	476 ± 51	562 ± 81	457 ± 35	500 ± 43

Data are expressed as mean ± SEM. LVESD, left ventricular end-systolic diameter; LVEDD, left ventricular end-diastolic diameter; LVESPW, left ventricular end-systolic posterior wall; LVEDPW, left ventricular end-diastolic posterior wall; LVSV, left ventricular stroke volume; LVFS, left ventricular fractional shortening; LVEF, left ventricular ejection fraction; BW, body weight. Data were analysed with two-way ANOVA followed by Sidak post hoc analysis. *p < 0.05 vs. Sham_Scr_ 4 wk; ^†^p < 0.05 vs. AV-Shunt_Scr_ 4 wk; ^‡^p < 0.05 vs. AV-Shunt_Gap27_ 4 wk; ^#^p < 0.05 vs. Sham_Scr_ 8 wk; ^§^p < 0.05 vs. AV-Shunt_Scr_ 8 wk.

### Chronic treatment with Gap27 improves cardiac function in AV-Shunt rats

**Figure 2:**
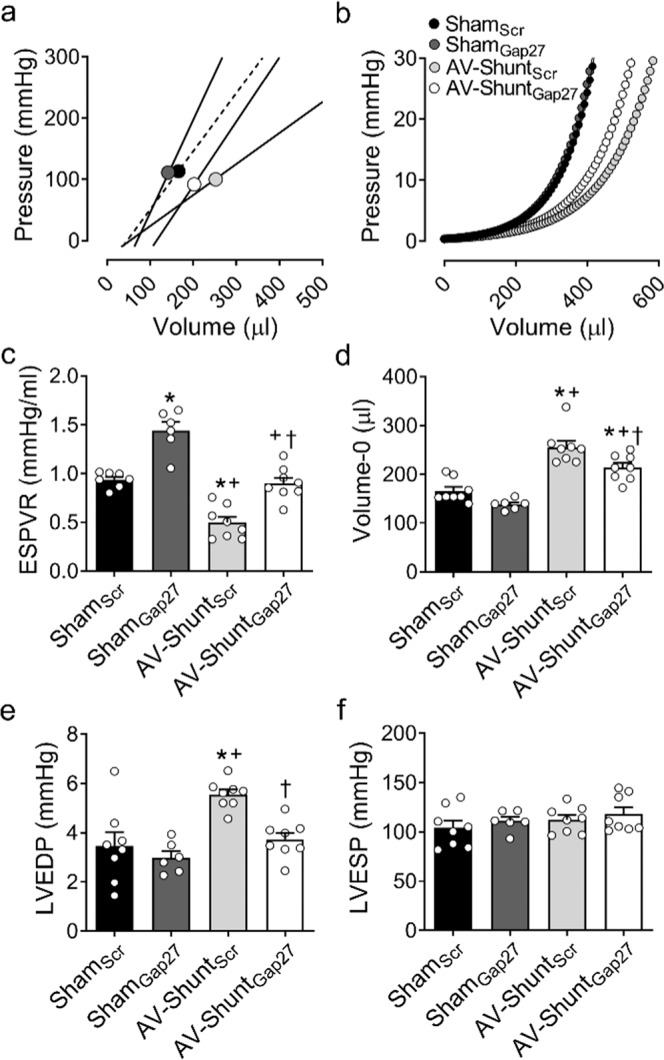
Effect of Gap27 on cardiac function in AV-Shunt rats. (**a**) End-systolic pressure-volume relationship (ESPVR) and (**b**) End-diastolic pressure-volume relationship (EDPVR) estimated by single-beat analysis from one Sham_Scr_ rat, one Sham_Gap27_ rat, one AV-Shunt_Scr_ rat and one AV-Shunt_Gap27_ rat at 8 wk. Note that Gap27 treatment significantly improves systolic and diastolic cardiac function measured by the slope (E_ES_) of ESPVR and b coefficient from EDPVR in AV-Shunt rats, respectively. In addition, Gap27 treatment improves ESPVR in Sham treated rats. (**c**,**d**) Summary data of the effects of Gap27 treatment on ESPVR and Volume at Pressure 0 mmHg (Volume-0) **(**predictors of EDPVR). (**e**,**f**). Summary data of the left ventricle end-diastolic pressure and left ventricle end-systolic pressure. Gap27 treatment reduced LVEDP in AV-Shunt rats, but not LVESP. Values are shown as mean ± SEM. Data were analysed with one-way ANOVA followed by Sidak post hoc analysis. *p < 0.05 vs. Sham_Scr_; ^+^p < 0.05 vs. Sham_Gap27_; ^†^p < 0.05 vs. AV-Shunt_Scr_. n = 8 animals per group.

Baseline PV-loop analysis at 8^th^ week showed that AV-Shunt_Scr_ rats displayed a significant increase in LVEDV and LVSV compared to Sham rats (Table 2). In addition, left ventricular end diastolic pressure (LVEDP) was significantly increased in AV-Shunt rats (5.6 ± 0.2 vs. 3.5 ± 0.5 mmHg; p < 0.05, AV-Shunt_Scr_ vs. Sham_Scr_) (Fig. 2b). Cardiac output and stroke work were also impaired in AV-Shunt_Scr_ rats compared to Sham rats (Table 2). Importantly, AV-Shunt rats that received Gap27 treatment showed a significant improvement in LVEDV (341.1 ± 16.6 vs. 467.1 ± 26.0 µl; p < 0.05, AV-Shunt_Gap27_ vs. AV-Shunt_Scr_), LVSV (239.7 ± 18.6 vs. 329.9 ± 22.4 µl; p < 0.05, AV-Shunt_Gap27_ vs. AV-Shunt_Scr_) and LVEDP (3.7 ± 0.3 vs. 5.6 ± 0.2 mmHg; p < 0.05, AV-Shunt_Gap27_ vs. AV-Shunt_Scr_) compared to scramble-treated AV-Shunt rats (Fig. 2 and Table 2). No significant effects of Gap27 on any other baseline hemodynamic parameters in AV-Shunt_Gap27_ rats were found (Fig. 2f and Table 2).

**Table 2:** Hemodynamic parameters from baseline pressure-volume loops.

	Sham_Scr_	Sham_Gap27_	AV-Shunt_Scr_	AV-Shunt_Gap27_
	(n = 8)	(n = 8)	(n = 8)	(n = 8)
HR (bpm)	366.3 ± 24.0	381.9 ± 12.7	327.8 ± 20.4	357.4 ± 18.5
LVESV (µl)	96.8 ± 12.8	105.6 ± 11.2	137.2 ± 14.3	101.4 ± 20.2
LVEDV (µl)	272.9 ± 18.9	257.4 ± 9.5	467.1 ± 26.0*†	341.1 ± 16.6†‡
LVSV (µl)	176.1 ± 13.6	151.8 ± 16.7	329.9 ± 22.4*†	239.7 ± 18.6†‡
CO (ml/min)	63.8 ± 5.7	57.6 ± 6.0	110.4 ± 13.4*†	86.0 ± 7.9
SW (mmHg/ml)	18.8 ± 2.4	17.0 ± 2.2	37.6 ± 4.1*†	28.0 ± 2.0†
LVEF (%)	64.8 ± 3.5	58.4 ± 4.9	70.7 ± 2.7	70.8 ± 5.6

Cardiac systolic and diastolic properties were evaluated by single beat analysis from PV-loops according to Klotz and Takeuchi^21,22^. AV-Shunt_Scr_ rats showed a significant reduction in the slope of the end systolic pressure-volume relationship (ESPVR:0.50 ± 0.06 vs. 0.94 ± 0.03 mmHg µl^−1^; p < 0.05, AV-Shunt_Scr_ vs. Sham_Scr_, respectively) and an increase in volume at pressure 0 mmHg (Volume-0: 255.7 ± 12.8 vs. 165.7 ± 8.5 µl; p < 0.05, AV-Shunt_Scr_ vs. Sham_Scr_, respectively) compared to Sham_Scr_ rats. Chronic Gap27 administration restored ESPVR and Volume-0 parameters in AV-Shunt_Gap27_ rats to values similar to those obtained in Sham_Scr_ rats (ESPVR: 0.90 ± 0.06 vs. 0.50 ± 0.06 mmHg/µl, p < 0.05, HF_Gap27_ vs. AV-Shunt_Scr_, respectively; Volume-0: 213.8 ± 9.4 vs. 255.7 ± 12.8 µl; p < 0.05, AV-Shunt_Gap27_ vs. AV-Shunt_Scr_) (Fig. 2a–d). The effects of Gap27 on ESPVR and Volume-0 in Sham rats are shown in Table 2.

Data are expressed as mean ± SEM. HR, Heart rate; LVESV, Left Ventricle End-Systolic Volume; LVEDV, Left Ventricle End-Diastolic Volume; SV, Stroke Volume; CO, Cardiac Output; SW, Stroke Work; LVEF, Left Ventricle Ejection Fraction. Data were analysed with one-way ANOVA followed by Sidak post hoc analysis. *p < 0.05 vs. Sham_Scr_; ^†^p < 0.05 vs. AV-Shunt_Scr_. n = 8 animals per group.

### Chronic Gap27 administration reduces arrhythmia incidence in AV-Shunt rats

**Figure 3:**
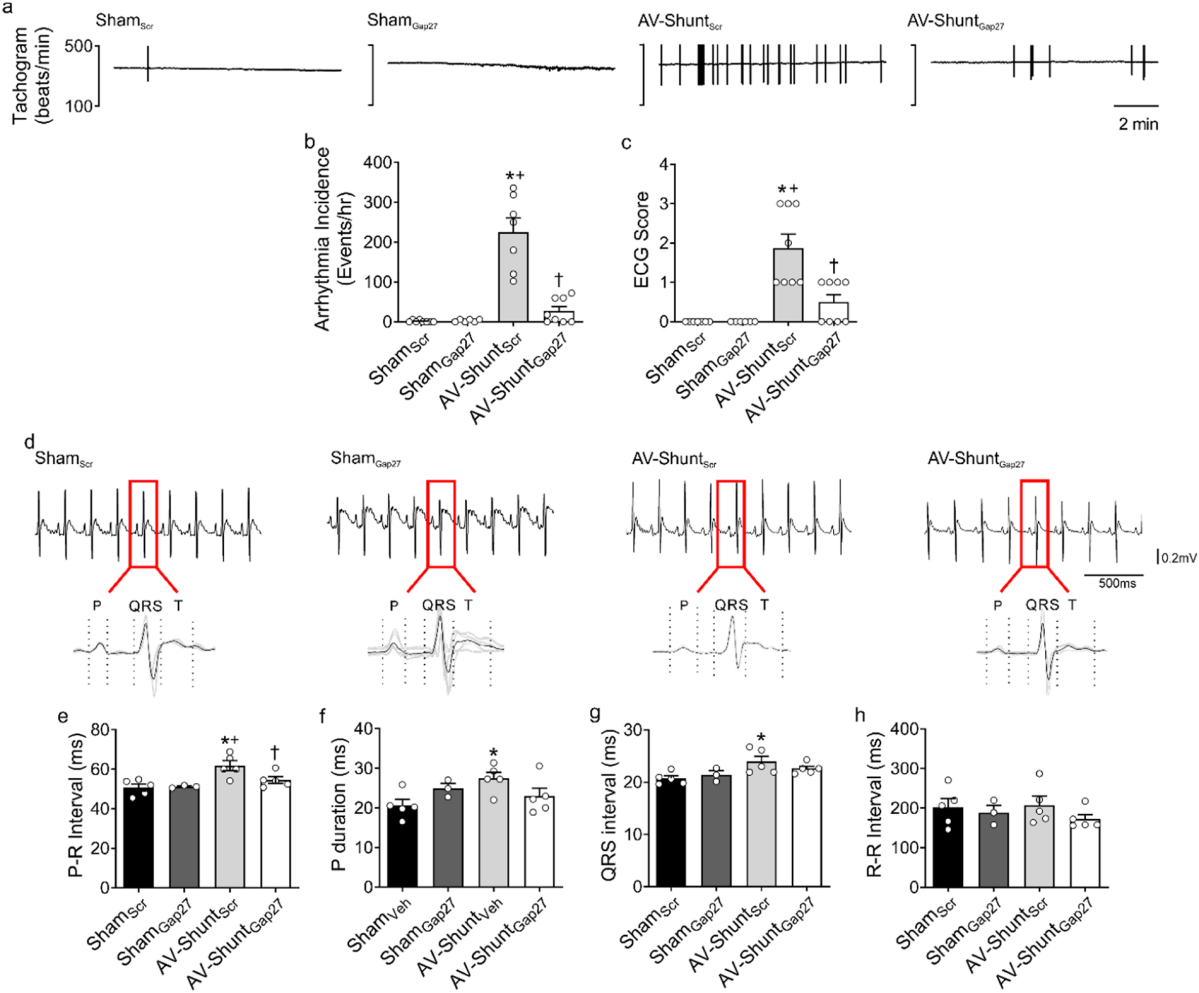
Gap27 administration improves cardiac arrhythmogenesis in AV-Shunt rats. (**a**) Representative tachograms from one Sham_Scr_ rat, one Sham_Gap27_ rat, one AV-Shunt_Scr_ rat and one AV-Shunt_Gap27_ rat obtained from EKG recordings. (**b**) Summary data show the incidence of ventricular premature beats (VPB). Note that Gap27 treatment reduces the arrhythmia incidence in AV-Shunt rats. (**c**) Summary data show the arrhythmic score. (**d**) Representative EKG recordings from one Sham_Scr_ rat, one Sham_Gap27_ rat, one AV-Shunt_Scr_ rat and one AV-Shunt_Gap27_ rat. Quantification of (**e**) PR interval, (**f**) P duration, (**g**) QRS interval and (**h**) RR interval. Values are shown as mean ± SEM. (**b**,**e–h**) Data were analysed with one-way ANOVA followed by Sidak post hoc analysis. **c** Data were analysed with Kruskal-Wallis test followed by Dunn post hoc analysis. *p < 0.05 vs. Sham_Scr_; +p < 0.05 vs. Sham_Gap27_; ^†^p < 0.05 vs. AV-Shunt_Scr_. n = 5–8 animals per group.

AV-Shunt_Scr_ rats showed an elevated number of arrhythmias compared to Sham_Scr_ rats (225.3 ± 35.2 vs. 1.5 ± 1.0 events/hr; p < 0.05, AV-Shunt_Scr_ vs. Sham_Scr_) (Fig. 3a,b). Likewise, arrhythmias severity was greater in AV-Shunt_Scr_ rats (1.9 ± 0.4 vs. 0.0 ± 0.0 a.u.; p < 0.05, AV-Shunt_Scr_ vs. Sham_Scr_) (Fig. 3C). Gap27 administration significantly reduced the arrhythmia incidence and severity in AV-Shunt rats (Arrhythmia incidence: 27.0 ± 11.1 vs. 225.3 ± 35.2 events/hr; p < 0.05, AV-Shunt_Gap27_ vs. AV-Shunt_Scr_; Arrhythmia Score: 0.5 ± 0.2 vs. 1.9 ± 0.4 a.u.; p < 0.05, AV-Shunt_Gap27_ vs. AV-Shunt_Scr_) (Fig. 3b,c). No significant effects of Gap27 on cardiac arrhythmogenesis were found in Sham rats (Fig. 3 and Table 3). Electrocardiogram (EKG) analysis showed that compared to Sham_Scr_ rats, AV-Shunt_Scr_ rats display increases in P wave duration (27.46 ± 1.6 vs. 20.6 ± 1.5 ms; p < 0.05, AV-Shunt_Scr_ vs. Sham_Scr_), PR interval duration (61.7 ± 2.5 vs. 50.8 ± 1.8 ms; p < 0.05, AV-Shunt_Scr_ vs. Sham_Scr_) and QRS interval duration (23.9 ± 1.0 vs. 20.7 ± 0.5 ms; p < 0.05, AV-Shunt_Scr_ vs. Sham_Scr_) (Fig. 3d–g). Gap27 treatment significantly reduced PR interval duration in AV-Shunt_Gap27_ rats compared to AV-Shunt_Scr_ rats (54.5 ± 1.7 vs. 61.7 ± 2.5 ms; p < 0.05, AV-Shunt_Gap27_ vs. AV-Shunt_Scr_) (Fig. 3e). R-R was not significantly different between all experimental conditions (Fig. 3h). EKG waves amplitudes are shown in Table 3.

**Table 3:** EKG parameters.

	Sham_Scr_	Sham_Gap27_	AV-Shunt_Scr_	AV-Shunt_Gap27_
	(n = 5)	(n = 5)	(n = 5)	(n = 5)
Heart Rate (bpm)	311.2 ± 33.2	327.2 ± 30.7	300.9 ± 28.1	355.5 ± 20.5
QTc (ms)	156.9 ± 3.9	166.6 ± 4.9	167.0 ± 4.8	164.7 ± 7.0
P Amplitude (µV)	65.7 ± 10.2	54.1 ± 13.1	65.5 ± 7.3	67.6 ± 13.1
Q Amplitude (µV)	–12.4 ± 8.8	–15.5 ± 0.7	–10.7 ± 6.0	–15.9 ± 9.9
R Amplitude (µV)	377.2 ± 38.0	272.1 ± 45.0	408.2 ± 34.0	443.6 ± 59.0
T Amplitude (µV)	170.8 ± 19.8	145.1 ± 10.2	153.8 ± 7.5	109.7 ± 20.0

Values are expressed as mean ± SEM. Data were analysed with Kruskal-Wallis test followed by Dunn post hoc analysis.

### Chronic treatment with Gap27 reverts cardiac remodeling in AV-Shunt rats

**Figure 4:**
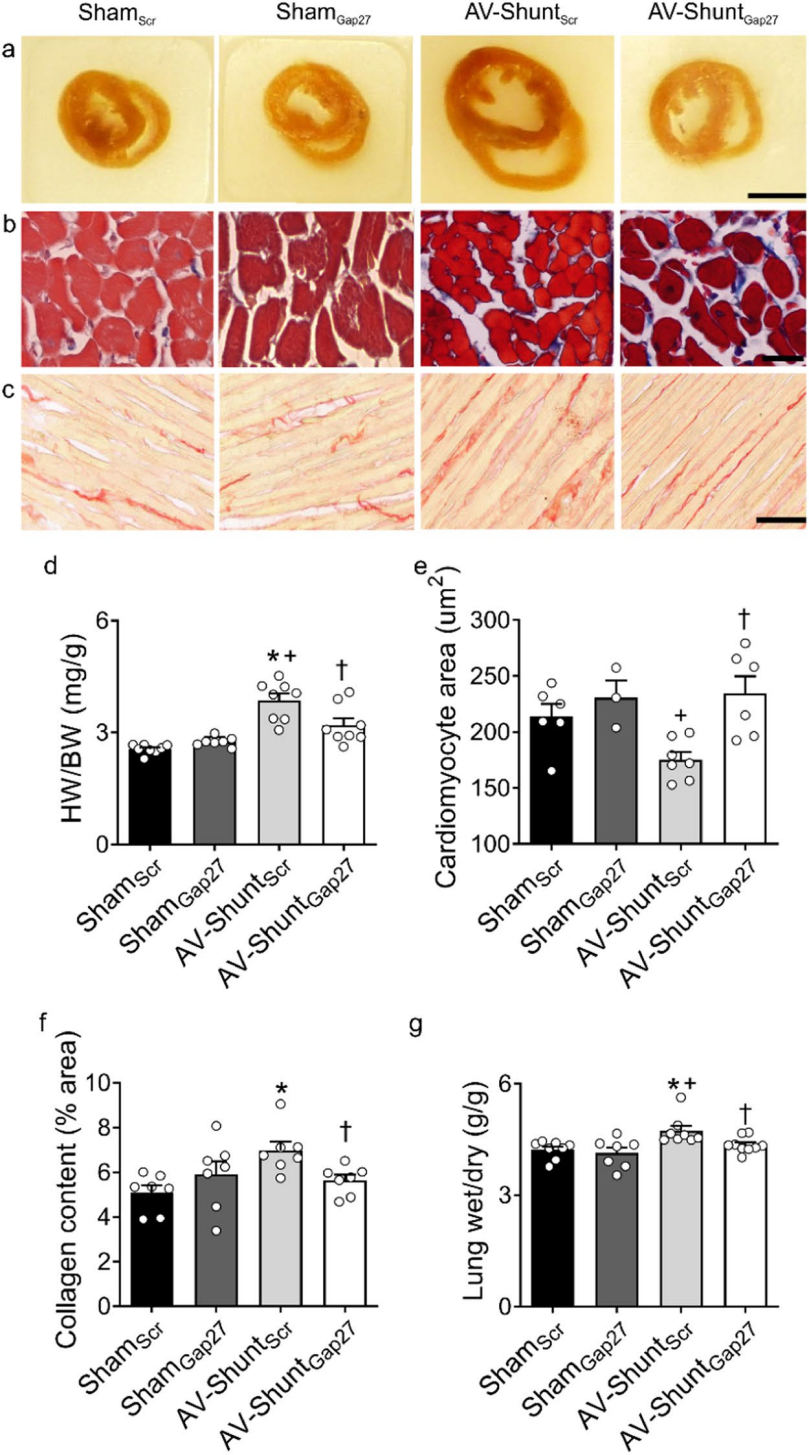
Gap27 reduces cardiac hypertrophy, fibrosis and pulmonary congestion in AV-Shunt rats. (**a**) Representative images of heart cross-sections obtained from one Sham_Scr_ rat, one Sham_Gap27_ rat, one AV-Shunt_Scr_ rat and one AV-Shunt_Gap27_ rat. Scale bar 5 mm. (**b**) Representative images of Masson’s trichome staining in heart cross-sections from one Sham_Scr_ rat, one Sham_Gap27_ rat, one AV-Shunt_Scr_ rat and one AV-Shunt_Gap27_ rat. Scale bar 25 µm. (**c**) Representative picrosirius red staining images in the left ventricle from one Sham_Scr_ rat, one Sham_Gap27_ rat, one AV-Shunt_Scr_ rat and one AV-Shunt_Gap27_ rat. Scale bar 250 µm. (**d**) Summary data of heart weight/body weight ratio. (**e**) Summary data show the cardiomyocyte cross-sectional area. (**f**) Summary data showing collagen content in the left ventricle. (**g**) Summary data show the lung congestion calculated as the lung wet/dry ratio. Values are shown as mean ± SEM. (**d–f)** Data were analysed with one-way ANOVA followed by Sidak post hoc analysis; (**g**) Data was analysed with Kruskal-Wallis test followed by Dunn post hoc analysis. *p < 0.05 vs. Sham_Scr_; +p < 0.05 vs. Sham_Gap27_; ^†^p < 0.05 vs. AV-Shunt_Scr_. n = 6–8 animals per group.

AV-Shunt_Scr_ rats showed cardiac hypertrophy and a reduction in cardiomyocyte area compared to Sham_Scr_ rats (Heart-to-body weight ratio, HW/BW: 3.9 ± 0.2 vs. 2.6 ± 0.1 mg/g; p < 0.05 AV-Shunt_Scr_ vs. Sham_Scr_; Cardiomyocyte area: 175.4 ± 6.7 vs. 214.0 ± 11.2 µm^2^; p < 0.05 AV-Shunt_Scr_ vs. Sham_Scr_) (Fig. 4a–e). Gap27 treatment significantly reduced cardiac hypertrophy index (HW/BW: 3.2 ± 0.2 vs. 3.9 ± 0.2 mg/g; p < 0.05 AV-Shunt_Gap27_ vs. AV-Shunt_Scr_) and increased cardiomyocyte area (175.4 ± 6.7 vs. 214.0 ± 11.2 µm^2^; p < 0.05 AV-Shunt_Gap27_ vs. AV-Shunt_Scr_) in AV-Shunt rats (Fig. 4d,e). No effects of Gap27 on heart weight was found in Sham rats (Fig. 4). In addition, the collagen content was increased in AV-Shunt_Scr_ rats compared to Sham_Scr_ rats (7.0 ± 0.4 vs. 5.1 ± 0.3%; p < 0.05 AV-Shunt_Scr_ vs. Sham_Scr_), and Gap27 treatment reduced collagen content in the hearts from AV-Shunt rats (5.6 ± 0.2 vs. 7.0 ± 0.4%; p < 0.05 AV-Shunt_Gap27_ vs. AV-Shunt_Scr_) (Fig. 4c,f). Also, AV-Shunt rats showed lung congestion compared to Sham rats (4.73 ± 0.14 vs. 4.23 ± 0.09 g/g; p < 0.05 AV-Shunt_Scr_ vs. Sham_Scr_) and Gap27 reduced pulmonary congestion index in AV-Shunt_Scr_ rats (Fig. 4g).

### Cx43 protein expression in the hearts of AV-Shunt rats are not affected by Gap27 administration

**Figure 5:**
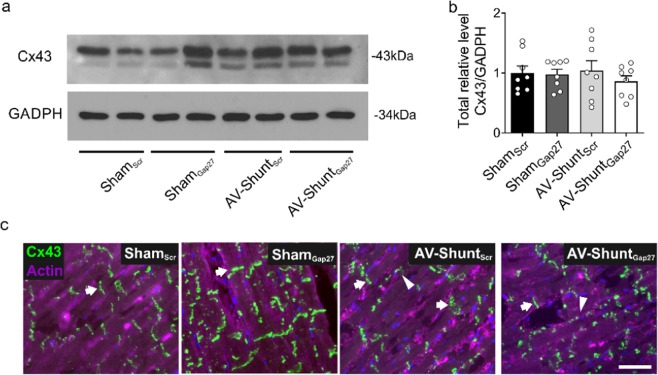
Effects of Gap27 on Cx43 protein expression in AV-Shunt rats. (**a**) Representative immunoblot of Cx43 expression in the rat left ventricle. (**b**) Densitometry analysis of Cx43 total relative expression. GADPH expression was analysed as the loading control. (**c**) Representative immunofluorescence of left ventricle labelled with anti-Cx43 antibody (green) and β-actin (pink) from one Sham_Scr_rat, one Sham_gap27_ rat, one AV-Shunt_Scr_ rat and one AV-Shunt_Gap27_ rat. Scale bar 25 µm. Note that in AV-Shunt_scr_ animals the patterns of Cx43 staining is lateralized compared to Sham_scr_ animals. Values are shown as mean ± SEM. Data were analysed with Kruskal-Wallis test followed by Dunn post hoc analysis. *p < 0.05 vs. Sham_Scr_. n = 8 per group.

AV-Shunt_Scr_ rats expressed similar protein levels of Cx43 compared to the ones observed in Sham_Scr_ rats (Fig. 5a,b). In addition, we found lateralization of Cx43 protein in AV-Shunt_Scr_ rats compared to Sham rats as evidenced by immunoreactivity in the lateral membrane of the cardiomyocyte, and this was not improved by Gap27 treatment (Fig. 5c).

## Discussion

In the present study, we sought to determine the effect of chronic administration of Cx43 mimetic peptide Gap27 on the progression of cardiac dysfunction and arrhythmogenesis in a high-output heart failure model induced by volume overload (i.e. AV-Shunt). The main findings of the present study are: i) Chronic Gap27 peptide administration decreases the progression of cardiac deterioration; ii) improves cardiac systolic and diastolic function; iii) reduces arrhythmia incidence and severity; and iv) reduces cardiac hypertrophy, adverse remodeling, and lung congestion in rats with high-output heart failure. Together, these findings strongly suggest that Cx43 plays a critical role in the progression of cardiac arrhythmogenesis and cardiac dysfunction in volume-overload heart failure.

Heart failure pathophysiology is characterized by a progressive decline in systolic (or contractile properties) and/or diastolic function (or passive properties)^2^. We found that 4 weeks after creation of AV-Shunt, animals display enlarged LV chambers in both systole and diastole that continues to increase up to 8 weeks post- AV-Shunt. Importantly, four weeks of chronic Gap27 peptide administration attenuated increases in systolic and diastolic chamber volumes in the AV-Shunt_Gap27_ group. This result suggests that Gap27 treatment has the potential to slow disease progression. Furthermore, AV-Shunt rats display both systolic and diastolic cardiac function impairment at 8 weeks post-induction, and chronic Gap27 treatment improved the contractile and passive properties of the heart in these animals. Interestingly, these effects were not exclusively observed in AV-Shunt animals since Sham rats treated with Gap27 also showed a mild improvement in systolic and diastolic cardiac function (Table 2). One plausible explanation for the beneficial effects of Gap27 could be a decrease in the “leak” of current through Cx43 hemichannels; which in turn would promote electrical conductivity primarily through gap junctions between cardiac cells and therefore reduce the formation of arrhythmic foci^11^. We speculate that in AV-Shunt rats^23^, the generation of cardiac arrhythmias could be related to the leakage of current from cardiomyocytes through hemichannels, creating a vicious cycle that promotes arrhythmogenesis and worsens cardiac function. Nevertheless, despite that mainly Gap27 peptide is used as a Cx43 blocker, it is important to mention that this peptide could block other connexin-based channels (i.e. Cx37) and also could affects Cx43 gap junction. Importantly, it has been shown that Cx43 hemichannel formation take place in the Golgi apparatus and then are transported directly to intercalated disc in cardiomyocytes *in vitro*^23,24^. Furthermore, Gap27 peptide lacks the TAT sequence which allow the peptide to easily permeate the cell membrane. However, we cannot completely rule out the possibility that Gap27 affect gap junctions. Then, it is possible to speculate that Gap27 peptide might decrease current leakage between cardiomyocytes, improving cardiac cell synchronicity and reducing arrhythmia incidence.

Cardiac arrhythmias are a strong predictor of morbidity and mortality in heart failure patients^6,25^, and play an especially prominent role in sudden cardiac death in this population^26,27^. In agreement with our previous findings, we observed a high incidence of cardiac arrhythmias in AV-Shunt rats^18,20^. AV-Shunt rats had a high arrhythmia score compared to Sham rats and chronic treatment with Gap27 markedly reduced the incidence and severity of the arrhythmic events. Recent studies by González *et al*.^16^ showed a similar salutary effect of Cx43 blockade on adrenergic-induced cardiac arrhythmias in a Duchenne muscular dystrophy model^16^. In addition, it has been shown that transplantation of Cx43-expressing embryonic cardiomyocytes (Cx43-eCMs) in myocardial infarction zone, markedly protects the heart from ventricular tachycardia induced by electrical pacing^13^. Together, these results suggest that controlling/improving current flow through gap junction channels instead of hemichannels in the failing heart reduces arrhythmia incidence.

Connexins are key proteins that allow the conduction of the electrical impulse between cardiomyocytes^10,12^. It has been described that one of the mechanisms related to cardiac arrhythmogenesis in various cardiovascular pathologies and animal models of heart diseases is the uncoupling of gap junctions and connexin hemichannel formation^12–16,28^. Some studies showed that minutes after the induction of ischemia in isolated rat hearts, a progressive increase in electrical uncoupling occurs, which is correlated with the formation of hemichannel structures^29^. Furthermore, alterations in the expression of Cx43 protein are associated with the generation of cardiomyopathies^13,30^. Knock-out mice lacking Cx43 protein die early, while partial knock-out mice, which express <50% of the protein, showed low survival due to cardiac dysfunction^30^. In contrast, overexpression of Cx43 in cardiac cells improves conductivity in infarcted hearts^31^. Here we showed no differences in the expression levels of  Cx43 in cardiac tissue from AV-Shunt rats compared to Sham rats and that Gap27 treatment improve cardiac arrhythmogenesis in AV-Shunt rats without changing Cx43 protein expression. In addition, we found a higher lateralization of Cx43 in cardiomyocytes from AV-Shunt animals which was not affected by Gap27. Recently, it has been proposed that Gap27 may work better on Cx43-S368 phosphorylated form^32^. Cx43-S368 is phosphorylated by PKC^33^. Under normal conditions in neonatal rat hearts, Cx43 display low phosphorylation in S368^34^. However, after 42 days of subcutaneous injections of isoprotenerol (a sympatho-mimetic agent), the phosphorylation of Cx43 and the expression of PKC in the left ventricle were increased, which was correlated with Cx43 lateralization^35^. These results suggest that increasing cardiac sympathetic drive enhances PKC activity and Cx43-S368 phosphorylation which may contribute to Cx43 lateralization in the heart. Then, is plausible to speculate that in HF conditions, a known condition where sympathoexcitation take place, cardiac Cx43 lateralization depends on S368 phosphorylation. Further studies are needed to completely address Cx43 phosphorylation status and the beneficial effect, if any, of administration of more specific mimetic peptide targeted against Cx43-S368 in HF pathophysiology.

Analysis of EKG waves can provide an indirect measure of cardiac electrical conductivity. In our experiments, we found several EKG abnormalities in AV-Shunt rats, including increases in P wave duration, the P-R interval, and the QRS interval. Importantly, chronic Gap27 administration normalized the observed EKG abnormalities in AV-Shunt rats. Prolonged P wave duration is a marker of electromechanical dysfunction which has been closely associated with an increase in atrial chamber size, atrial fibrillation, and death in cardiovascular diseases^36,37^. Also, it is thought that prolonged P wave duration reflects the presence of arrhythmogenic substrates both in the atrium and in the ventricle. In support of this notion, a linear relationship between the fibrotic index and the duration of the P wave has been observed^38^. The P-R interval denotes the time from the beginning of atrial depolarization to the onset of ventricular depolarization^39^. A prolonged P-R interval can be attributed to delayed conduction through the AV node, through the atrial tissue, or through the Purkinje system. More importantly, a prolonged P-R interval is associated with a higher risk of atrial fibrillation and mortality^39^. Importantly, our findings showed that AV-Shunt rats displayed increases in P-R interval duration compared to Sham rats and that Cx43 mimetic peptide Gap27 significantly reduces PR interval duration. However, our results did not discard the effect of Gap27 on Cx43 gap junction formation in the intercalated disc, which could contribute to decrease P-R duration in AV-Shunt animals.

Patients with heart failure often present with a prolonged QRS interval^40,41^. Prolonged QRS interval is indicative of delayed electrical conduction, which can result in both intra- and interventricular mechanical desynchrony. This desynchrony leads to inefficient contraction and an increase in energy demand ultimately leading to structural remodeling of the heart^42–44^. Cardiac hypertrophy in conjunction with structural remodeling and reduction of cardiac efficiency results in an increase in wall stress and electrical remodeling like Cx43 re-localization from the intercalated disc to the lateral membrane. Together these perpetuate the desynchrony and remodeling cycle contributing to deterioration of cardiac function in heart failure^45^. Accordingly, ventricular desynchrony in heart failure patients is associated with poor outcomes. Interestingly, heart failure patients who have successfully responded to resynchronization therapy, to reverse desynchrony, shown signs of reverse remodeling (i.e. LVEDV reductions) along with improve survival^43,46^. We found that Gap27 treatment reduces LVEDV, hypertrophic index, and collagen content in the hearts of AV-Shunt rats. Therefore, Gap27 treatment may improve cardiac synchrony in AV-Shunt rats. However, we did not specifically test this hypothesis in this study. Moreover, it has been shown that Cx43 expression is not restricted only to cardiomyocytes in the heart but also is expressed in fibroblast^47^. Therefore, is it possible that our results regarding cardiac remodeling could be associated to effects on cardiomyocytes and also to fibroblast in the heart. Our data suggests that Gap27 treatment improves cardiac electrical conductivity in both the atria and the ventricle. Future studies should address whether Gap27 treatment improves cardiac synchrony.

In summary, we found that chronic treatment with Gap27 prevents the progression of cardiac dysfunction, improves electrical and cardiac remodeling and reduces arrhythmogenesis in high-output heart failure. Our results suggest that Gap27 may have salutary effects in the setting of heart failure as a therapeutic tool for the treatment of cardiac arrhythmogenesis and cardiac function impairment.

## Materials and Methods

### High-output heart failure model

Adult male Sprague-Dawley rats (320 ± 9 g) (n = 24) underwent arterio-venous shunt (AV-Shunt) surgery to induce overload volume to the heart. This protocol was performed under sterile conditions, keeping the animal anaesthetized with isoflurane (5% for induction, 1.5% for maintenance in O_2_). The postoperative management consisted in the administration of 1 mg/kg of ketoprofen (s.c.), 5 mg/kg of enrofloxacin (s.c.) and 5 ml of sterile physiological saline (i.p.), together with the topical administration of 2% lidocaine hydrochloride. Rats with Sham surgery (n = 16) were handled under the same conditions. At 4th week post-shunt, the degree of cardiac failure was evaluated by M-mode echocardiography on an anaesthetized animal under isoflurane (5% for induction, 1.5% for maintenance in O_2_). Rats that fulfil following criteria were considered in this study: i) Ejection fraction (EF) ≥ 50; ii) End diastolic volume (EDV) and iii) stroke volume (SV) ≥ mean+2 standard deviation (SD) fold changes relative to Sham_Scr_ rats of its experimental series^18–20^. Animals were handled in accordance with standards set by the National Institutes of Health guidelines for the Care and Use of Laboratory Animals. All experimental protocols were approved by the Ethics Committee of the Pontificia Universidad Católica de Chile (protocol ID #170710022).

### Chronic Gap27 peptide administration

At fourth week post-shunt a second surgery for the implantation subcutaneous of an osmotic mini-pump (2.5 µl/hr, 2ML4, ALZET) containing the Cx43 mimetic peptide Gap27 (Sham_Gap27_ and AV-Shunt_Gap27_) (ANASPEC, amino acid sequence SRPTEKTIFII) or Scrambled Gap27 (Sham_Scr_ and AV-Shunt_Scr_) (ANASPEC, amino acid sequence RQLIITSFIPT) at a concentration of 1 µg/kg diluted in sterile physiological saline were implanted for 28 days^48^. The surgery was performed under general anesthesia with isoflurane (5% for induction, 1.5% for maintenance flush in O_2_) and a postoperative management consisted in the administration of 1 mg/kg of ketoprofen (s.c.), 5 mg/kg of enrofloxacin (s.c.) and 5 ml of sterile physiological saline solution (i.p.), together with the topical administration of 2% lidocaine hydrochloride.

### Transthoracic echocardiography

Cardiac deterioration was evaluated by M-mode echocardiography (SONOACE R3, Samsung, USA) under general anesthesia with isoflurane (5% for induction, 1.5% for maintenancein O_2_). M-mode views were recorded at the level of mid-papillary muscle. Left ventricle end systolic diameters (LVESD) and left ventricle end diastolic diameters (LVEDD) were determined from M-mode images using averaged measurements from 3 consecutive cardiac cycles according to the American Society of Echocardiography. Left ventricle end systolic volume (LVESV) and end diastolic volume (LVEDV) were calculated by Teichholz method:

$$\begin{array}{c}LVESV=\frac{7\times LVES{D}^{3}}{2.4+LVESD}\\ LVEDV=\frac{7\times LVED{D}^{3}}{2.4+LVEDD}\end{array}$$

Other parameters such as ejection fraction, fractional shortening and stroke volume were calculated^18,48^:

$$\begin{array}{c}LVEF=100\times \frac{LVEDV-LVESV}{LVEDV}\\ LVFS=\frac{LVEDD-LVESD}{LVEDD}\\ LVSV=LVEDV-LVESV\end{array}$$

### Invasive hemodynamic parameters

Rats were anaesthetized with a mixture of 800 mg/kg urethane (SIGMA, USA) and 40 mg/kg α-chloralose (SIGMA, USA). A transducer catheter (SPR-869, MILLAR INSTRUMENTS) was introduced into the left ventricle through the right carotid. Cardiac left ventricle pressure-volume (PV) loops were recorded at steady state. Measurements were calibrated by injecting a hypertonic saline bolus (30% wt/vol NaCl) to determine conductance, and relative volume units were converted absolute using the cuvette calibration method. The LV end-of-systole volume (LVESV), LV end-diastolic volume (LVEDV), LV end-systole pressure (LVESP), LV end-diastolic pressure (LVEDP), ejection volume (SV), cardiac output (CO) and ejection fraction (EF) were obtained from 10–15 representative loops. Volume at pressure 0 and End-Systolic Pressure-Volume Relationship (ESPVR) were used as indicators of diastolic and systolic function, respectively. These analyses were calculated by a mathematical algorithm from a single beat obtained from pressure-volume curves according to Klotz *et al*.^21^ for V-0 and Takeushi *et al*.^22^ for ESPVR. All recordings were sampled to 1 kHz and were analyzed using LABCHART7 Pro v7.2 software (ADINSTRUMENTS)^18,19,21,22,49^.

### Arrhythmia Scoring and electrocardiography (EKG) analysis

2-lead EKG was recorded in anaesthetized rats with a mixture of 800 mg/kg urethane (SIGMA, USA) and 40 mg/kg α-chloralose (SIGMA, USA). The incidence of arrhythmias was determined as arrhythmic events in one hour of recording. The arrhythmia score was calculated according to scores previously used in Curtis *et al*. (1988–2013)^50,51^ and Miller *et al*.^51^, based on the kind and duration of the arrhythmic event, and using the definitions established in the Lambeth Conventions II^52^. A ventricle premature beat (VPB) was defined as a ventricular electrical complex (complete electrical event: QRS, RS, QRST or RST) that is different in shape (voltage and/or duration, i.e., height and/or width) from the preceding (non-VPB) ventricular complex. Ventricle Tachycardia (VT) was defined as a sequence of a less 4 VPB consecutive. Ventricle Fibrillation (VF) was defined as a sequence of a minimum of 4 consecutive ventricular complexes without intervening diastolic pauses, which peak-peak interval and the height vary without a pattern. In addition, for waves analysis, the EKG signal was processing with the Stationary Wavelet Transform Denoising 1D tool from MATLAB software. Then, the signal denoised was analyzed with EKG plugin from LABCHART 7 Software (ADINSTRUMENTS). Parameters such as the P wave, PR interval, QRS interval, QTc (Bazzett), T wave, RR interval were calculated. All recordings were sampled to 1 kHz^49–51^.

### Histological analysis

After rats were euthanized, hearts were removed and arrested in diastole with KCl 1 M (SIGMA, USA). Immediately, hearts were fixed with 4% paraformaldehyde and were stored in PBS + 0.1% azide until embedded in paraffin. Transversal sections of 5 µm thickness were stained with modified Masson’s trichrome stain (without hematoxylin) (DIAPATH, Italy) and picrosirius red (DIAPATH, Italy). The deposition of interstitial collagen was quantified by using IMAGEJ software (NIH Software, USA) in images acquired at 40×(NIKON ECLIPSE E400). Per cent fibrosis was expressed as the ratio of the fibrotic area into total left ventricle area.

### Immunostaining

Cardiac sections of 20 µm thick were cut in a cryostat at −20 °C. Tissue were incubated with blocking solution with 5% gelatin from cold-water fish skin, 0.5% Triton X-100 and Phosphate Buffer Saline (137 mM NaCl, 2.7 mM KCl, 10 mM Na_2_HPO_4_, 1.8 mM KH_2_PO_4_). Then were incubated with primary rabbit antibody against Connexin-43 (71–0700, THERMOFISHER) at a 1:100 dilution in blocking solution, followed by incubation with the secondary anti-rabbit antibody conjugated with Alexafluor-488 at a 1:1,000 and Alexa Fluor-594 phalloidin at a 1:1,000 diluted in blocking solution. Slices were mounted in Vectashield with DAPI (VECTOR Laboratories). The immunostaining was visualized in a high-resolution fluorescent microscope (LEICA, Germany) and images were acquired at 40×.

### Western blot

Left ventricle tissue samples were homogenized in RIPA buffer (SIGMA, USA) supplemented with 1% protease inhibitor (SIGMA, USA) plus 1% of phosphatases inhibitors (PHOSPHOSTOP SIGMA, USA), then centrifuged at 12,000 rpm for 30 min at 4 °C and the supernatant was stored at −80 °C. The protein concentration was determined by a BCA assay (PIERCE), following the manufacturer’s specifications. 50 ug of proteins were separated by SDS-PAGE under reducing conditions on gels of 12% of acrylamide and electrophoretically transferred to PVDF membranes (MILLIPORE). The electrophoretic run was performed in the Tris-glycine buffer for 2 hours at 100 V constant voltage and the transfer was performed in cold, for 2 hours at 300 mA constant. The membrane was blocked with 5% non-fat milk in Tris Saline Buffer (20 mM Tris base, pH 7.2, 0.3 M NaCl) with 0.05% tween20. The membrane was immunoblotted with the primary antibodies against Connexin-43 (71–0700, THERMOFISHER) and GADPH (sc-32233, SANTACRUZ) at a 1:1000 and 1:2,000 dilution in 5% milk in TBS, respectively. Next, were incubated with secondary antibody conjugated with HRP (Anti-rabbit: 074–1506, KPL; Anti-mouse: 074-1806, KPL) at a dilution of 1:2000 in blocking solution plus 0.1% tween20. Proteins were visualized using ECL Western Blotting Detection kit (PIERCE). Each membrane was exposed to high sensitivity film (BIOMAX MR Film of Carestream, KODAK) and was revealed with development and fixation solution (DENTUS, AGFA, USA). Finally, the films were scanned, and the images were analyzed with the software image studio lite version 5.2 (LI-COR BIOSCIENCE, USA). The relative amount of proteins was calculated as the intensity ratio of the band of the protein of Cx43 versus the intensity of the band corresponding to GADPH.

### Statistical analysis

The results were shown as mean ± standard error of the mean (SEM). Differences between groups were assessed with one or two-way ANOVA, accordingly to matrix data, followed by Sidak´s posthoc comparisons for parametric distribution. While for non-parametric data, was analyzed with Kruskal-Wallis test followed by Dunn post hoc test. Differences between two groups were assessed t-test for parametric distribution or Mann-Whitney test for nonparametric distribution. A probability value of p < 0.05 was considered statistically significant. All tests were performed by GRAPHPAD PRISM software (La Jolla, CA, USA, version 7.0).

## Data Availability

All data generated/analyzed during this study are included in this article (including the Supplemental Material).

## References

[1] Zafrir B (2018). Prognostic implications of atrial fibrillation in heart failure with reduced, mid-range, and preserved ejection fraction: a report from 14 964 patients in the European Society of Cardiology Heart Failure Long-Term Registry. Eur Heart J.

[2] Kemp CD, Conte JV (2012). The pathophysiology of heart failure. Cardiovasc Pathol.

[3] Rewiuk K (2011). Epidemiology and management of coexisting heart failure and atrial fibrillation in an outpatient setting. Pol Arch Med Wewn.

[4] Lip GY (2016). European Heart Rhythm Association/Heart Failure Association joint consensus document on arrhythmias in heart failure, endorsed by the Heart Rhythm Society and the Asia Pacific Heart Rhythm Society. Europace.

[5] Lee DS (2011). A systematic assessment of causes of death after heart failure onset in the community: impact of age at death, time period, and left ventricular systolic dysfunction. Circ Heart Fail.

[6] Zile MR (2010). Mode of death in patients with heart failure and a preserved ejection fraction: results from the Irbesartan in Heart Failure With Preserved Ejection Fraction Study (I-Preserve) trial. Circulation.

[7] Santangeli P, Rame JE, Birati EY, Marchlinski FE (2017). Management of Ventricular Arrhythmias in Patients With Advanced Heart Failure. J Am Coll Cardiol.

[8] Coronel R (2013). Electrophysiological changes in heart failure and their implications for arrhythmogenesis. Biochim Biophys Acta.

[9] Nielsen MS (2012). Gap junctions. Compr Physiol.

[10] Leybaert L (2017). Connexins in Cardiovascular and Neurovascular Health and Disease: Pharmacological Implications. Pharmacol Rev.

[11] Retamal MA (2015). Diseases associated with leaky hemichannels. Front Cell Neurosci.

[12] Severs NJ (2004). Remodelling of gap junctions and connexin expression in heart disease. Biochim Biophys Acta.

[13] Kalcheva N (2007). Gap junction remodeling and cardiac arrhythmogenesis in a murine model of oculodentodigital dysplasia. Proc Natl Acad Sci USA.

[14] Roell W (2007). Engraftment of connexin 43-expressing cells prevents post-infarct arrhythmia. Nature.

[15] Seidel T, Salameh A, Dhein S (2010). A simulation study of cellular hypertrophy and connexin lateralization in cardiac tissue. Biophys J.

[16] Gonzalez JP, Ramachandran J, Xie LH, Contreras JE, Fraidenraich D (2015). Selective Connexin43 Inhibition Prevents Isoproterenol-Induced Arrhythmias and Lethality in Muscular Dystrophy Mice. Sci Rep.

[17] Xue J (2019). Connexin 43 dephosphorylation contributes to arrhythmias and cardiomyocyte apoptosis in ischemia/reperfusion hearts. Basic Res Cardiol.

[18] Andrade DC (2017). Exercise training improves cardiac autonomic control, cardiac function, and arrhythmogenesis in rats with preserved-ejection fraction heart failure. J Appl Physiol (1985).

[19] Del Rio R (2017). Carotid Body-Mediated Chemoreflex Drive in The Setting of low and High Output Heart Failure. Sci Rep.

[20] Toledo C (2017). Cardiac diastolic and autonomic dysfunction are aggravated by central chemoreflex activation in heart failure with preserved ejection fraction rats. J Physiol.

[21] Klotz S (2006). Single-beat estimation of end-diastolic pressure-volume relationship: a novel method with potential for noninvasive application. Am J Physiol Heart Circ Physiol.

[22] Takeuchi M (1991). Single-beat estimation of the slope of the end-systolic pressure-volume relation in the human left ventricle. Circulation.

[23] Basheer WA (2017). GJA1-20k Arranges Actin to Guide Cx43 Delivery to Cardiac Intercalated Discs. Circ Res.

[24] Basheer W, Shaw R (2016). The “tail” of Connexin43: An unexpected journey from alternative translation to trafficking. Biochim Biophys Acta.

[25] Giannoni A (2008). Clinical significance of chemosensitivity in chronic heart failure: influence on neurohormonal derangement, Cheyne-Stokes respiration and arrhythmias. Clin Sci (Lond).

[26] Luu M, Stevenson WG, Stevenson LW, Baron K, Walden J (1989). Diverse mechanisms of unexpected cardiac arrest in advanced heart failure. Circulation.

[27] Vaduganathan M, Patel RB, Shah SJ, Butler J (2016). Sudden cardiac death in heart failure with preserved ejection fraction: a target for therapy?. Heart Fail Rev.

[28] Patel PM (2001). Altering ventricular activation remodels gap junction distribution in canine heart. J Cardiovasc Electrophysiol.

[29] Beardslee MA (2000). Dephosphorylation and intracellular redistribution of ventricular connexin43 during electrical uncoupling induced by ischemia. Circ Res.

[30] Danik SB (2004). Modulation of cardiac gap junction expression and arrhythmic susceptibility. Circ Res.

[31] Greener ID (2012). Connexin43 gene transfer reduces ventricular tachycardia susceptibility after myocardial infarction. J Am Coll Cardiol.

[32] Cotter ML (2019). The lipidated connexin mimetic peptide SRPTEKT-Hdc is a potent inhibitor of Cx43 channels with specificity for the pS368 phospho-isoform. Am J Physiol Cell Physiol.

[33] Schulz R (2015). Connexin 43 is an emerging therapeutic target in ischemia/reperfusion injury, cardioprotection and neuroprotection. Pharmacol Ther.

[34] Nassal MM (2016). Phosphorylation at Connexin43 Serine-368 Is Necessary for Myocardial Conduction During Metabolic Stress. J Cardiovasc Electrophysiol.

[35] Viczenczova, C. *et al*. Myocardial connexin-43 is upregulated in response to acute cardiac injury in rats. *Can J Physiol Pharmacol* **95**:911–919, 10.1139/cjpp-2016-0680 (2017). 10.1139/cjpp-2016-0680 28459162

[36] Ariyarajah V, Mercado K, Apiyasawat S, Puri P, Spodick DH (2005). Correlation of left atrial size with p-wave duration in interatrial block. Chest.

[37] Goyal SB, Spodick DH (2001). Electromechanical dysfunction of the left atrium associated with interatrial block. Am Heart J.

[38] Tiffany Win T (2015). Associations of electrocardiographic P-wave characteristics with left atrial function, and diffuse left ventricular fibrosis defined by cardiac magnetic resonance: The PRIMERI Study. Heart Rhythm.

[39] Cheng S (2009). Long-term outcomes in individuals with prolonged PR interval or first-degree atrioventricular block. JAMA.

[40] Kashani A, Barold SS (2005). Significance of QRS complex duration in patients with heart failure. J Am Coll Cardiol.

[41] Iuliano S (2002). QRS duration and mortality in patients with congestive heart failure. Am Heart J.

[42] Fauchier L (2002). Interventricular and intraventricular dyssynchrony in idiopathic dilated cardiomyopathy: a prognostic study with fourier phase analysis of radionuclide angioscintigraphy. J Am Coll Cardiol.

[43] Breidthardt T (2007). QRS and QTc interval prolongation in the prediction of long-term mortality of patients with acute destabilised heart failure. Heart.

[44] Matsumoto K (2011). Reverse remodelling induces progressive ventricular resynchronization after cardiac resynchronization therapy ‘from vicious to virtuous cycle’. Eur J Echocardiogr.

[45] Willenbrock R (1999). Angiotensin inhibition and atrial natriuretic peptide release after acute volume expansion in rats with aortocaval shunt. Cardiovasc Res.

[46] Bleeker GB, Bax JJ, Steendijk P, Schalij MJ, van der Wall EE (2006). Left ventricular dyssynchrony in patients with heart failure: pathophysiology, diagnosis and treatment. Nat Clin Pract Cardiovasc Med.

[47] Tayal B (2017). Interaction of Left Ventricular Remodeling and Regional Dyssynchrony on Long-Term Prognosis after Cardiac Resynchronization Therapy. J Am Soc Echocardiogr.

[48] Tarzemany R, Jiang G, Jiang JX, Larjava H, Hakkinen L (2017). Connexin 43 Hemichannels Regulate the Expression of Wound Healing-Associated Genes in Human Gingival Fibroblasts. Sci Rep.

[49] Andrade DC (2019). Ablation of brainstem C1 neurons improves cardiac function in volume overload heart failure. Clin Sci (Lond).

[50] Curtis MJ (2013). The Lambeth Conventions (II): guidelines for the study of animal and human ventricular and supraventricular arrhythmias. Pharmacol Ther.

[51] Curtis MJ, Walker MJ (1988). Quantification of arrhythmias using scoring systems: an examination of seven scores in an *in vivo* model of regional myocardial ischaemia. Cardiovasc Res.

[52] Miller LE, Hosick PA, Wrieden J, Hoyt E, Quindry JC (2012). Evaluation of arrhythmia scoring systems and exercise-induced cardioprotection. Med Sci Sports Exerc.

